# Hypereosinophilia with Concurrent Venous Thromboembolism: Clinical Features, Potential Risk Factors, and Short-term Outcomes in a Chinese Cohort

**DOI:** 10.1038/s41598-020-65128-4

**Published:** 2020-05-20

**Authors:** Yecheng Liu, Xu Meng, Jun Feng, Xianliang Zhou, Huadong Zhu

**Affiliations:** 1 Department of Emergency, Peking Union Medical College Hospital, Peking Union Medical College and Chinese Academy of Medical Sciences, Beijing, 100730 China; 2 Department of Cardiology, Fuwai Hospital, National Center for Cardiovascular Disease, Chinese Academy of Medical Sciences and Peking Union Medical College, Beijing, 100037 China; 3 Department of Hematopathology, Peking Union Medical College Hospital, Peking Union Medical College and Chinese Academy of Medical Sciences, Beijing, 100730 China

**Keywords:** Thromboembolism, Haematological diseases

## Abstract

Idiopathic hypereosinophilia (IHE) and hypereosinophilic syndrome (HES) are benign haematological disorders. Studies have suggested that venous thromboembolism (VTE) is a rare but sometimes fatal complication of hypereosinophilia; however, data are limited. We retrospectively analysed clinical features and short-term outcomes of 63 consecutive patients (82.5% men; mean age, 40.92 ± 10.89 years) with IHE or HES with concurrent VTE from January 1998 through December 2018. Risk factors for pulmonary embolism (PE) were explored by multivariate logistic analysis. DVT and/or PE was detected by imaging in all patients. Independent risk factors for PE were a body mass index of >24.1 kg/m^2^ (odds ratio [OR]: 5.62, 95% confidence interval [CI]: 1.21–26.13, P = 0.028), peak absolute eosinophil count of >6.3 × 10^9^/L (OR: 5.55, 95% CI: 1.292–23.875, P = 0.021), and >13.9-month duration of hypereosinophilia (OR: 4.51, 95% CI: 1.123–18.09, P = 0.034). All patients were treated with corticosteroids and anticoagulants. The short-term hypereosinophilia remission rate was 100%; no recurrent VTE or major bleeding was observed. Hypereosinophilia is a potential risk factor for VTE. PE in patients with IHE/HES and DVT is associated with a higher body mass index, higher peak absolute eosinophil count, and longer duration of hypereosinophilia. Corticosteroids and anticoagulants provided effective short-term control of hypereosinophilia and VTE.

## Introduction

Hypereosinophilia (HE) encompasses a diverse group of disorders characterised by peripheral blood eosinophilia (>1.5 × 10^9^/L) with or without a primary cause (such as haematologic, autoimmune, parasitic, or allergic disease)^1^. Idiopathic HE (IHE) is HE without a primary aetiology or organ damage. When HE without a primary aetiology induces organ damage, it is termed hypereosinophilic syndrome (HES). Damage to various organs has been reported in patients with IHE and HES, most commonly dermal, lung, cardiovascular, and gastrointestinal system damage. Venous thromboembolism (VTE) is a rare complication of HE^1,2^.

Previous studies have focused on thrombus formation in patients with IHE/HES^3^. A few studies have suggested that approximately 25% of patients with HES develop thromboembolic complications, with an associated mortality rate of 5% to 10%^2^. Many types of thrombosis have been reported, including mural thrombus of the heart, inferior vena cava (IVC) thrombosis, superficial venous thrombosis, portal thrombosis, deep venous thrombosis (DVT) with or without pulmonary embolism (PE), cerebral arteriolar and venous thrombosis, and intracardiac thrombi^2,4–10^. Among these, DVT with PE is a rare but often fatal complication of HES^11^. Because most reported cases lacked primary aetiologies and traditional risk factors for VTE, we hypothesised that HE may cause development of VTE.

Because of the rarity and nonspecific clinical features of IHE/HES, available data on thromboembolism in patients with these disorders are limited and sporadic. Information on the clinical features and outcomes of these conditions is also limited^11^. To better understand the thromboembolic complications of HE, this case series summarises the patient demographics, clinical features, and short-term clinical outcomes in patients with IHE/HES and concurrent VTE. We also explored potential risk factors for thrombotic events among patients with HE without traditional risk factors, which may provide valuable evidence for clinical practice.

## Materials and Methods

### Patients

Consecutive patients with IHE/HES and concurrent VTE (DVT and/or PE) who were hospitalised at Peking Union Medical College Hospital from 1 January 1998 to 31 December 2018 were identified from electronic medical records. The inclusion criteria were as follows: (1) age of 18 to 60 years at the time of VTE diagnosis, (2) presence of symptomatic and imaging-confirmed VTE during or within 1 month before hospitalisation, (3) no history of VTE, (4) onset of VTE after a chronic course of HE (≥6 months), and (5) Autar DVT risk assessment scale (2002) score of ≤5^12^. Patients with thrombophilia, including protein C or S deficiency, antithrombin III deficiency, antiphospholipid antibodies, or hyperhomocysteinaemia were excluded^12^. The enrolled patients were divided into a PE group and non-PE (NPE) group according to the presence of PE.

The study complied with the Declaration of Helsinki (World Medical Assembly) and its amendments and was approved by the Ethics Committee of Peking Union Medical College Hospital. The requirement for written informed consent was waived by the Ethics Committee because this was a retrospective study.

### Definitions and data collection

HE was defined as an absolute eosinophil count (AEC) of >1.5 × 10^9^/L in two separate examinations on different days and/or pathologic confirmation of tissue HE^13,14^. IHE was defined as HE without evidence of a specific cause. HES refers to IHE, as defined above, combined with eosinophil-mediated organ damage and/or dysfunction^15^. To exclude parasitic infections, all patients underwent three separate stool sample examinations for ova and parasites on different days. Each patient underwent detection of antibodies to *Toxoplasma*, flukes, nematodes, and hydatid tapeworms to rule out common parasitic infections. All patients were also tested for protein C or S deficiency, antithrombin III deficiency, antiphospholipid antibodies, activated protein C resistance, lupus anticoagulant, abnormal tumour marker levels, and hyperhomocysteinaemia to screen for congenital and acquired thrombophilia. Genetic testing of the JAK2 V617F mutation was also performed in patients with hepatic and/or portal vein thrombosis to exclude primary haematologic thrombophilia^16^. PE was classified as massive PE, submassive PE, or low-risk PE according to the guidelines of the American Heart Association and American College of Chest Physicians^17^.

Two trained researchers retrospectively extracted data on the patients’ demographic characteristics, medical history, clinical manifestations, blood test results, organ involvement, and treatments. Pathology-confirmed tissue HE combined with structural and/or functional abnormalities of organs on imaging, laboratory, and/or electrophysiological tests was defined as organ involvement after exclusion of other factors. Potential duplicate records with matching birth year, sex, and clinical features were excluded. Laboratory examinations included routine blood tests and screening tests for the underlying aetiology of HE. Involvement of organs (e.g., skin, muscle, lymph nodes, lung, heart, gastrointestinal tract, and/or kidney) was routinely evaluated with imaging examinations, with pathological assessment in some cases. Involvement of peripheral nerves was evaluated with electromyography. All participants underwent ultrasound evaluation of veins in the upper and lower extremities, vena cava, and portal vein and its branches. PE was identified by multidetector row computed tomography pulmonary angiography or V/Q scanning^18^. The body mass index (BMI) was calculated as kg/m^2^.

### Therapies and short-term outcomes

Corticosteroids (CS) (e.g., prednisone, prednisolone, and methylprednisolone) are first-line drugs in the treatment of IHE and HES^1^. Patients were initially given a medium (0.6–0.8 mg/kg of prednisone) to high (0.8–1.0 mg/kg of prednisone) CS dose, which was gradually decreased to the maintenance dose. Glucocorticoid pulse therapy is the short-term use of CS at a dosage equal to 0.5 to 1.0 g per day of hydrocortisone, which is always gradually decreased to a high dose of CS.

Treatment for DVT includes anticoagulation and implantation of an IVC filter. Both heparin combined with warfarin and novel oral anticoagulants are first-line choices for anticoagulant therapy. Unfractionated or low-molecular-weight heparin (LMWH) was prescribed for the initial treatment of VTE until 5 days after the international normalised ratio reached 2 to 3. Warfarin was started on the same day as heparin or LMWH to maintain the international normalised ratio at 2 to 3. Oral dabigatran or edoxaban was initiated after 5 days of heparin or LMWH. Loading doses of oral apixaban or rivaroxaban were administered at the onset of VTE and/or PE. IVC filters were considered in patients with recurrent PE and in those with complications (such as bleeding) that required cessation of anticoagulation. Management of PE depended on risk stratification. Treatment for low-risk PE was similar to that for DVT. In patients with submassive and massive PE, therapeutic strategies, especially the decision to conduct reperfusion treatment, were determined on the basis of the risk score for PE, haemodynamics, and bleeding risks^19^. Other treatment principles were in accordance with accepted recommendations^19,20^. All therapeutic strategies were determined and adjusted by experienced physicians according to recognised principles.

Short-term outcomes were evaluated by determination of the AEC and incidence of major bleeding and recurrent VTE 3 to 6 months after discharge as shown in the follow-up records at the clinic. The most recent data were extracted when more than one record was found. Major bleeding was defined in accordance with the International Society on Thrombosis and Haemostasis major bleeding criteria^21^. Recurrent VTE was defined as new-onset symptoms confirmed by imaging examinations, as fatal PE, or as death of unknown cause with the inability to rule out PE.

### Data analysis

Data were analysed with IBM SPSS Statistics Version 21.0 (IBM Corp., Armonk, NY, USA). Continuous variables are expressed as mean ± standard deviation, and categorical variables are expressed as number and percentage. Differences between groups were evaluated with the chi-square test or Fisher’s exact test for categorical variables and the independent Student’s t test for continuous variables. Multivariate logistic analysis was conducted to detect the independent risk factors for PE. In the multivariate logistic regression analysis, we included independent parameters with a P value of <0.10 in the comparisons between the PE and NPE groups as well as items that correlated with development of PE based on our clinical experience and previous studies. The forward LR method of multivariate logistic regression was used. The entry and removal levels were 0.05 and 0.10, respectively. Items with a P value of <0.05 were considered independent risk factors for PE in the multivariate logistic regression analysis. Parameters with an odds ratio (OR) of >1 were considered risk factors, whereas parameters with an OR of <1 were considered protective factors. The BMI, peak AEC, and course of HE were transformed into dichotomous variables at cutoff values determined with the receiver operating characteristics (ROC) curve. Finally, age, sex, BMI of >24.1 kg/m^2^, peak AEC of >6.2 × 10^9^/L, >13.9-month duration of HE, and presence of HES with lung involvement were included in the multivariate logistic regression analysis. A two-sided P value of <0.05 was considered to indicate statistical significance.

## Results

### Clinical features of patients

In total, 63 patients were enrolled, including 52 men (82.5%) and 11 women (17.5%). The mean age at diagnosis was 40.92 ± 10.89 (range, 23–59) years. The mean duration of HE until diagnosis of VTE was 15.49 ± 12.21 (range, 6–84) months. Thirty-one patients had a medical history of CS use for treatment of HE. The most common comorbidities were hypertension (n = 11, 17.5%) and diabetes mellitus (n = 8, 12.7%). Twenty-four (28.10%) patients had a history of allergies. The most common manifestations of multiorgan HE involvement were dermatologic (29/63, 46.0%), followed by pulmonary (28/63, 44.4%) and gastrointestinal (26/63, 41.3%). The most common symptom of DVT was lower extremity oedema (31/59, 52.5%), including 29 symmetrical and 2 asymmetrical cases. DVT in the remaining patients was found because of concurrent PE or numbness and/or weakness of the lower extremities. Among patients with PE, the most common symptom was haemoptysis (14/31, 45.2%), followed by thoracic pain (10/31, 32.3%), cough (9/31, 29.0%), and dyspnoea (8/31, 32.3%).

Table 1 shows the baseline AEC, proportion of eosinophils, white blood cell count, haemoglobin level, and platelet count at admission as well as the peak AEC during the course of HE. The peak AEC ranged from 1.7 to 18 × 10^9^/L, with a mean of 6.86 ± 3.64 × 10^9^/L.

**Table 1:** Baseline characteristics of patients.

Variables	All (n = 63)	PE group (n = 31)	NPE group (n = 32)	P value*
Age at diagnosis, years	40.92 ± 10.89	41.45 ± 11.32	40.41 ± 10.63	0.71
Course of HE, months	15.49 ± 12.21	17.70 ± 14.49	13.35 ± 9.22	0.16
Female	11 (17.5%)	4 (12.9%)	7(21.9%)	0.35
BMI, Kg/m^2^	24.47 ± 2.10	25.55 ± 1.79	23.43 ± 1.85	**<0.01**
**Medical histories**				
Using CS before thrombosis	31(49.2%)	6(19.4%)	25(78.1%)	**<0.01**
Hypertension	11(17.5%)	3(9.7%)	8(25.0%)	0.11
CAD	1(3.1%)	0(0%)	1(3.1%)	0.32
Diabetes mellitus	8(12.7%)	3(9.7%)	5(15.6%)	0.48
Hyperlipemia	0(0%)	0(0%)	0(0%)	—
Allergy	24(38.10%)	12(44.4%)	12(37.5%)	0.59
Smoking	35(55.6%)	20(64.5%)	15(46.9%)	0.16
Drinking	34(54.0%)	15(48.4%)	19(59.4%)	0.38
**Blood routine test**				
AEC on admission,10^9^/L	4.01 ± 3.29	5.13 ± 3.46	2.93 ± 2.76	**<0.01**
Peak AEC,10^9^/L	6.86 ± 3.64	8.46 ± 4.05	5.31 ± 2.38	**<0.01**
WBC on admission,10^9^/L	13.98 ± 5.54	15.96 ± 6.07	12.05 ± 4.26	**<0.01**
Hb on admission, g/L	140.78 ± 18.71	143.718 ± 16.37	137.948 ± 20.58	0.22
PLT on admission, 10^9^/L	166.89 ± 99.18	161.10 ± 90.98	172.50 ± 107.69	0.65
Eosinophil infiltration	45(71.4%)	19(42.2%)	26(57.8%)	0.08
**Organ involvements**				
Skin	15(25.4%)	7(22.6%)	9(28.1%)	0.61
Heart	9(14.3%)	5(16.1%)	4(12.5%)	0.68
Lung	27(42.9%)	10(32.3%)	17(53.1%)	0.09
Kidney	1(3.1%)	0(0%)	1(3.1%)	0.32
Gastrointestinal	14(22.2%)	8(25.8%)	6(18.8%)	0.50
Lymph gland	14(22.2%)	6(19.4%)	8(25.0%)	0.59
Muscle	4(9.5%)	2(6.5%)	4(12.5%)	0.41
Peripheral nerve systems	14(22.2%)	9(29.0%)	5(15.6%)	0.20

Data are presented as mean ± standard deviation or n (%).

AEC, absolute eosinophil count; BMI, body mass index; CAD, coronary artery disease; CS, corticosteroid; HE, hypereosinophilia; Hb, haemoglobin; PE, pulmonary embolism; NPE, non-pulmonary embolism; PLT, platelet; WBC, white blood cell.

Organ infiltration by eosinophils was confirmed on pathologic examination of tissue biopsy in 45 patients, resulting in a diagnosis of IHE in 28.6% (18/63) and HES in 71.4% (45/63) of all patients. In total, 56 tissue biopsies were examined in 50 (79.4%) patients, including 15 biopsies of lymph nodes, 14 of skin, 9 of the gastrointestinal tract, 8 of subcutaneous nodules, 7 of muscle, and 3 of lung. Eosinophilic infiltration of organs was observed in 45 patients (13 in lymph nodes, 11 in skin, 7 in the gastrointestinal tract, 6 in muscle, 6 in subcutaneous nodules, and 2 in lung). All organ involvement identified by biopsy and/or imaging examinations is shown in Table 1.

DVT and/or PE was detected with imaging in all patients. More than 90% of patients had DVT (59/63, 93.7%), and 45.8% (27/59) of these had concurrent PE. The other patients (4/63, 6.3%) had PE without imaging evidence of DVT. Concurrent thrombosis of abdominal visceral veins was common on Doppler ultrasound. The most commonly involved vein was the portal vein (16/63, 25.4%), followed by the hepatic vein (12/63, 19.0%), superior mesenteric vein (8/63, 12.7%), splenic vein (4/63, 6.3%), inferior mesenteric vein (2/63, 3.2%), and renal vein (1/63, 1.6%). Brachial vein thrombosis was detected in a male patient; no mural thrombus was observed in the heart chambers. IVC thrombosis was detected in two patients (2/63, 3.2%), including a 46-year-old man with Budd–Chiari syndrome.

### Potential risk factors for PE development

In total, 31 cases of PE were identified, including 1 massive PE, 1 submassive PE, and 29 low-risk PE. We divided the cohort into two groups according to the presence of PE: the PE group (31/63, 49.2%) and the NPE group (32/63, 40.8%). The clinical features of both groups are shown in Table 1. Compared with patients in the NPE group, patients in the PE group had a significantly longer duration of HE, higher AEC on admission, higher peak AEC, higher white blood cell count, and lower prevalence of prior CS use.

**Figure 1:**
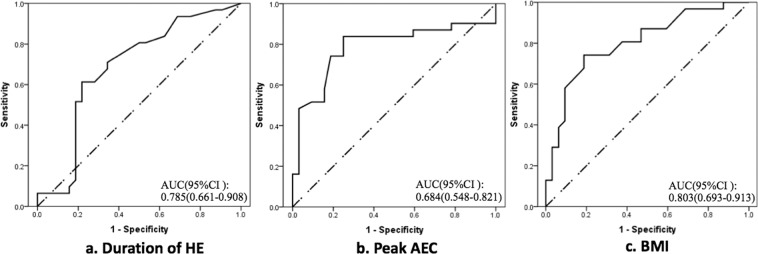
Receiver operating characteristics curves and areas under the curve of three parameters related to development of pulmonary embolism. (**a**) shows that the cutoff value for the duration of hypereosinophilia was 13.9 months (sensitivity: 61.3%, specificity: 78.1%, P = 0.012). (**b**) indicates that the cutoff value for the peak absolute eosinophil count was 6.2 × 10^9^/L (sensitivity: 83.9%, specificity: 75.0%, P < 0.01). (**c**) shows that the cutoff value for the body mass index was 24.1 kg/m^2^ (sensitivity: 80.6%, specificity: 62.5%, P < 0.01).

Because interactions were possible among a patient’s history of CS use, peak AEC, AEC on admission, and white blood cell count, only a peak AEC of >6.2 × 10^9^/L (determined with ROC curve) was included in the further logistic regression analysis. As shown in Table 2, the multivariate logistic regression analysis identified the following independent risk factors for PE in our cohort: BMI of >24.1 kg/m^2^ (OR: 5.62, 95% confidence interval [CI]: 1.21–26.13, P = 0.028) (see ROC curve, Fig. 1a), peak AEC of >6.2 × 10^9^/L (OR: 5.55, 95% CI: 1.292–23.875, P = 0.021) (see ROC curve, Fig. 1b), and >13.9-month duration of HE (OR: 4.51, 95% CI: 1.123–18.09, P = 0.034) (see ROC curve, Fig. 1c).

**Table 2:** Multivariate logistic regression analysis of risk factors for developing pulmonary embolism.

Variables	OR (95% CI)	P value
BMI	5.62 (1.21–26.13)	0.028
Peak AEC > 6.2 × 10^9^/L	5.55 (1.292–23.875)	0.021
Duration of HE > 13.9 months	4.51 (1.123–18.09)	0.034

AEC, absolute eosinophil count; BMI, body mass index; CI, confidence interval; HE, hypereosinophilia; OR, odds ratio.

### Treatments

All patients were prescribed CS for treatment of IHE or HES. Thirty-one (49.2%) patients had a medical history of CS use before the occurrence of DVT and/or PE. Fifty-two patients (82.5%) had taken prednisone, eight (12.7%) had taken prednisolone, and three (4.8%) had taken methylprednisolone. The dosages of prednisolone and methylprednisolone were converted to equivalent dosages of prednisone using ratios of 1:1 and 4:5, respectively. The initial dose of CS ranged from 30 to 80 mg of prednisone per day (mean, 53.33 ± 15.13 mg/day). Steroid pulse therapy was administered in 11 (17.5%) patients with severe organ damage (lung, n = 6; heart, n = 4; and kidney, n = 1) with a dose of 0.5 to 1.0 g of hydrocortisone per day by injection. The duration of steroid pulse treatments ranged from 3 to 5 days, after which patients were treated with high-dose CS (10 received 60 mg and 1 received 80 mg of prednisone per day).

Anticoagulant therapy was started in all patients after the diagnosis of DVT and/or PE, and the course of treatment was 3 months after restoration of the AEC. Only one patient underwent systemic thrombolytic therapy to treat obstructive shock caused by massive PE. Because only patients with first-time PE were included, none received an IVC filter during hospitalisation.

### Short-term outcomes

Short-term outcomes of HE and VTE were evaluated according to predefined criteria (Table 3). The mean follow-up period was 4.2 ± 0.9 months. Sixty patients had medical records at our outpatient department during the predefined time window (3–6 months after discharge), and data regarding the AEC were acquired among all of these patients. All patients achieved remission of HE, and >90% had complete remission. Forty-one patients underwent ultrasonography of the bilateral lower extremity veins, and none of them had concomitant new-onset DVT. The other patients reported no symptoms indicating new-onset DVT or PE. Although most patients with PE did not undergo re-examination with computed tomography pulmonary angiography or V/Q scanning, all patients’ clinical status was confirmed. No major bleeding was identified. In the patient with massive PE, the obstructive shock was completely corrected and the clinical symptoms had resolved at follow-up.

**Table 3:** Short-term outcomes of hypereosinophilia and deep vein thrombosis.

Variables	Evaluation criterions	Number	Portion
**HE**			
Complete remission	AEC < 0.5ple^9^/L	59/60	98.3%
Partial remission	0.5tial r < AEC < 1.5tia^9^/L and lower than on admission	1/60	1.7%
Non-remission	AEC > 1.5-re^9^/L or not lower than on admission	0/60	0%
**VTE**			
Recurrent VTE	The new onset symptoms confirmed by image examinations, or fatal PE, or death for unknown cause where PE cannot be ruled out.	0/60	0%
Major bleeding	(1)Fatal bleeding, and/or symptomatic bleeding in a critical area or organ, and/or (2) bleeding causing a fall in hemoglobin level ≥ 20 g L^−1^ or more, or leading to transfusion ≥ 2units of whole blood or red cells.	0/60	0%

AEC, absolute eosinophil count; HE, hypereosinophilia; DVT, deep venous thrombosis.

## Discussion

HES, a rare disorder with undefined pathogenesis, is considered the most severe nonmalignant form of eosinophil-related disease because it can cause damage to various organs. VTE is a rare complication of HES, with only occasional reports. Previous studies have summarised many risk factors for VTE, including both inherited and acquired^20^. Identification and elimination of these risk factors is important for prevention and treatment of VTE. Because no additional aetiology of VTE can be identified in many patients with IHE/HES, we hypothesised that the high level of eosinophils in the blood and tissues increases the risk of VTE in these patients. Therefore, in this study, we selected 63 patients with HE of unknown cause (18 IHE and 45 HES) with occurrence of VTE at least 6 months after onset of HE. The proportion of patients with HES in our cohort may be much higher than that in the general HE population because patients with HES are more prone to be admitted with severe organ damage, whereas patients with IHE are easily overlooked because they lack obvious clinical symptoms.

Several points support the hypothesis that high levels of circulating eosinophils may cause VTE. First, none of the patients in our cohort had traditional risk factors for DVT or PE with the exception of age and weight^12,20^. We also used an Autar DVT risk assessment scale (2002) score of ≤5 to ensure that all enrolled patients had a very low risk of DVT at the time of DVT and/or PE occurrence. Because they lacked traditional risk factors, these patients also underwent a series of tests to screen for thrombophilia during hospitalisation; patients with any type of thrombophilia were excluded. Second, all patients were symptomatic at the time of VTE diagnosis and had no history of VTE, which increases the probability that they had new-onset VTE. Third, all VTEs were identified after the onset of HE. Additionally, although cases of VTE in patients with HE are sporadic, hundreds have been reported because of the rarity, severity, and poor prognosis of such cases^3–6,22,23^. Uderhardt *et al*.^24^ also found a significant association between elevated plasma levels of eosinophilic cationic protein (an eosinophil-activation marker) and new-onset atherothrombotic events.

Several potential mechanisms may explain thrombus formation secondary to HE. First, eosinophils play an important role in activation and regulation of blood coagulation. Previous research confirmed the expression of tissue factor (TF) and autonomous TF-dependent generation of thrombin by eosinophils^24–26^. Active eosinophils can induce release of platelet-activating factor, which in turn activates leukocytes, endothelial cells, and platelets and releases TF^26^. Eosinophils can also provide a procoagulant phospholipid surface supporting TF-mediated thrombin generation. Eosinophilic cationic protein, a protein particle contained in eosinophils, stimulates thrombus formation by binding heparin and neutralising its anticoagulating effects, shortening the coagulation time of normal plasma via interactions with factor XII^27^. Second, endothelial injury is a potential mechanism of thrombosis development. Pathologic changes of Löffler endocarditis and endomyocardial fibrosis may induce the formation of an intracardiac mural thrombus in patients with HES^28–30^. Major basic protein is another important particle in eosinophils. Previous studies have suggested that major basic protein-induced direct toxic damage to microvascular endothelial cells may cause microthrombi in patients with HE. However, whether similar damage exists in venous endothelial cells need further study, especially pathological evaluation of veins with DVT^31,32^. Finally, inflammation plays a key role in venous thromboembolic disease, and eosinophils can regulate local inflammatory responses^33,34^. Activated eosinophils release various substances, including cytokines, chemokines, lipid mediators, and cytotoxic granule proteins, which can initiate and promote local inflammation when they accumulate in blood and tissues^33^. Previous studies have indicated that the inflammatory response is amplified by CD40 ligand expressed on blood eosinophils, which is related to initiation and progression of thrombosis^35,36^.

PE is the third most common cause of cardiovascular death, after heart attack and stroke^37^. Most PEs are derived from DVTs of the lower extremities. Nearly half of DVTs may induce silent PE^19^. A literature analysis conducted by Maino *et al*.^3^ showed that 16.7% of 36 patients with sporadic HES with venous thrombosis had PE. In our cohort, 45.8% of patients with DVT had concurrent PE, mostly low-risk PE. Although this ratio might have been underestimated because of the possibility of silent PE, it is higher than the reported prevalence^3^. This higher prevalence may have resulted from admission bias because the severe condition of patients with PE made them more likely to be identified and hospitalised in our centre.

The multivariate logistic analysis showed that a peak AEC of >6.3 × 10^9^/L, >13.9-month duration of HE, and BMI of >24.1 kg/m^2^ were independent risk factors for PE development among patients with IHE/HES and DVT. Although excess body weight is a well-recognised risk factor for VTE, a high AEC and long duration of HE have rarely been considered as risk factors. Our results suggest that lowering the eosinophil count and shortening the duration of HE with effective therapeutic methods may be essential in preventing the occurrence of thrombotic events. A higher eosinophil count and longer disease duration may indicate more severe effects on coagulation, endothelial damage, and inflammatory responses. However, there are no studies on this subject, and further exploration is needed to elucidate the exact mechanisms.

The first-line treatment of idiopathic HE is administration of CS, which are also effective in treating HES^1,38^. In our cohort, all patients’ AEC decreased and 98.3% achieved complete remission after treatment with CS. Standard anticoagulation treatments also effectively prevent the recurrence of VTE, and no severe bleeding events were observed during the short-term follow-up. There is no published information about the long-term outcomes of CS and anticoagulant therapy in patients with idiopathic eosinophilia (including HES) and VTE. Whether anticoagulation prevents progression of thrombotic complications in IHE is also unclear. Prospective randomised controlled trials will be helpful to establish more precise and individualised treatments for these patients.

## Limitations

This study has several limitations. First, this was a retrospective study; therefore, selection bias, such as admission bias, was inevitable. Second, some cases of HES may have been misdiagnosed as IHE because of lack of pathologic evidence of eosinophilic infiltration. Third, this study included patients who were hospitalised over a long time span (from 1998 to 2018), and more than half of the patients were lost to follow-up. Therefore, long-term outcomes were not analysed.

## Conclusions

In summary, VTE is a rare complication of IHE and HES. We propose that HE is a potential risk factor for VTE. The occurrence of PE in our cohort was associated with a higher BMI, higher peak AEC, and longer duration of HE, suggesting that early treatment to lower AEC may help prevent thrombotic complications. CS and anticoagulants showed satisfactory effects in controlling HE and VTE in the short-term observation.
